# Effect of Chest Resistance and Expansion Exercises on Respiratory Muscle Strength, Lung Function, and Thoracic Excursion in Children with a Post-Operative Congenital Diaphragmatic Hernia

**DOI:** 10.3390/ijerph19106101

**Published:** 2022-05-17

**Authors:** Alshimaa R. Azab, Walid Kamal Abdelbasset, Saud M. Alrawaili, Abbas Elbakry A. Elsayed, Mohammed Ibrahim Hajelbashir, FatmaAlzahraa H. Kamel, Maged A. Basha

**Affiliations:** 1 Department of Health and Rehabilitation Sciences, College of Applied Medical Sciences, Prince Sattam Bin Abdulaziz University, Al-Kharj 11942, Saudi Arabia; walidkamal.wr@gmail.com (W.K.A.); s.alrawaili@psau.edu.sa (S.M.A.); 2 Department of Physical Therapy for Pediatrics, Faculty of Physical Therapy, Cairo University, Giza 12613, Egypt; 3 Department of Physical Therapy, Kasr Al-Aini Hospital, Cairo University, Giza 12613, Egypt; 4 Department of Pediatrics, College of Medicine, Prince Sattam Bin Abdulaziz University, Al-Kharj 11942, Saudi Arabia; ae.elsayed@psau.edu.sa (A.E.A.E.); abukhalid218@gmail.com (M.I.H.); 5 Department of Pediatrics, Faculty of Medicine, Al-Azhar University, Assuit 71524, Egypt; 6 Department of Physical Therapy for Surgery, Faculty of Physical Therapy, Cairo University, Giza 12613, Egypt; dralzahraafatma@gmail.com; 7 Department of Physical Therapy, College of Medical Rehabilitation, Qassim University, Buraidah 51452, Saudi Arabia; bashamaged@gmail.com; 8 Department of Physical Therapy, El-Sahel Teaching Hospital, General Organization for Teaching Hospitals and Institutes, Cairo 11697, Egypt

**Keywords:** diaphragmatic hernia, chest resistance exercise, chest expansion exercise, respiratory muscle strength, lung functions, thoracic excursion

## Abstract

Background. Congenital diaphragmatic hernia (CDH) is a life-threatening condition with long-term complications including respiratory tract infections, respiratory muscle weakness, and abnormal lung functions. This study was designed to ascertain the effects of chest resistance and chest expansion exercises on respiratory muscle strength, lung function, and chest mobility in children with post-operative CDH. Methods. This randomized controlled clinical study was conducted in the outpatient physiotherapy clinic at Prince Sattam bin Abdulaziz University. Thirty-two children with CDH aged 10–14 years between May 2020 and February 2021 were randomly allocated to the study group (*n* = 16) and the control group (*n* = 16). The control group underwent a usual chest physiotherapy program; however, the study group underwent a 12-week chest resistance exercise combined with chest expansion exercise in addition to usual chest physiotherapy, with three sessions per week. Respiratory muscle strength, lung function, and thoracic excursion were assessed pre- and post-treatment. Results. Using the 2 × 2 repeated ANOVA, significant time × group interactions were detected in favor of the study group, FVC (F = 4.82, 95% CI = −15.6 to −0.97, *p* = 0.005, and η^2^ = 0.16), FEV1 (F = 4.54, 95% CI = −11.99 to −2.8, *p* ˂ 0.001, and η^2^ = 0.14), PImax (F = 5.12, 95% CI = −15.71 to −5.3, *p* ˂ 0.001, and η^2^ = 0.15), and thoracic excursion (F = 4.41, 95% CI = −2.04 to −0.16, *p* = 0.036, and η^2^ = 0.17). Conclusions. Concurrent chest resistance and expansion exercises may improve respiratory muscle strength, lung function, and thoracic excursion in children with post-operative CDH. The study findings suggest that concurrent chest and chest expansion exercises be part of an appropriate pulmonary rehabilitation program in children with a history of CDH.

## 1. Introduction

Congenital diaphragmatic hernia (CDH) is a potentially fatal birth defect that occurs in 1 in 3000 live births [1]. It is caused by a lack of diaphragm muscularization during embryogenesis, resulting in an incomplete or absent diaphragm [2], which leads to the presence of abdominal content in the thoracic cavity, interfering with normal lung development [3,4]. The CDH may exist as an isolated lesion or as part of a syndrome with a higher prevalence among males than females, and often occurs as a unilateral condition, frequently on the left side [5].

Survival rates in children with CDH have improved in recent decades due to advances in surgical and neonatal treatment [6]. The surviving children may suffer from long-term complications such as impairments in lung growth (lung hypoplasia), cardiovascular disorders, pulmonary hypertension, gastrointestinal problems, and recurrent occurrences of lower respiratory tract infection [7,8]. In addition, survivors with CDH had significant lung dysfunction, including decreased total lung volume due to abnormal antenatal lung growth, peripheral airway obstruction, decreased specific compliance of the respiratory system, and decreased chest wall mobility compared to normal peers [9]. Furthermore, when compared to normal peers after surgical correction of diaphragmatic abnormality or mechanical ventilation, respiratory muscle strength was significantly lower in children with CDH due to decreased lung volume [10].

However, there are many ways to treat children who have CDH, such as taking corticosteroids before they are born to help their lungs grow, or after they are born using conventional mechanical ventilation (CMV) or surgery to repair the diaphragm [11,12,13]. There are many ways to help kids with chest and respiratory problems, such as respiratory muscle training [14], chest physiotherapy [15], and aerobic exercise training [16]. One of the physical therapy techniques used to treat chest disorders via encouraging the normal alignment of respiratory muscles with respiratory rhythm is chest resistance exercise through applying resistance to the sternal and coastal areas [17]. In contrast, chest expansion exercise is a whole-body exercise that merges active movements of the trunk and limbs with deep breathing. This type of exercise can enhance chest and intercostal space mobility and can also decrease the stiffness in connective tissue [18].

To our knowledge, no studies have been conducted to evaluate the effects of chest resistance exercise on inspiratory muscle strength, lung function, and thoracic excursion in children with a post-operative congenital diaphragmatic hernia. Our study, therefore, was designed to investigate the effects of chest resistance exercise and chest expansion exercise on respiratory muscle strength, lung function, and chest mobility in those children.

## 2. Materials and Methods

### 2.1. Study Design

This randomized controlled clinical trial was conducted at the outpatient physiotherapy clinic at Prince Sattam bin Abdulaziz University between May 2020 and February 2021. The local institutional review board of the physiotherapy department granted ethical clearance, No.: RHPT/020/056, and the study was registered on ClinicalTrials.gov, ID: NCT04900649. All procedures were fulfilled in agreement with the ethical standards of the 1964 Declaration of Helsinki and its updates. The outlines of the study were clearly demonstrated to children and their families before starting the study procedures. Parents were asked to sign a written consent form once they approved their children’s participation in the study.

### 2.2. Participants

Thirty-two children with post-operative CDH of both sexes joined this study. They were recruited from the pediatric surgical department, King Khalid Hospital, Maternity Hospital, and other referral hospitals in Al-Kharj, Saudi Arabia. The inclusion criteria were: children of ages 10 to 14 years, BMI of 20 to 25 kg/m^2^, and considered at high risk for CDH because they developed respiratory distress in the first days of life. CDH was corrected surgically immediately after birth, and the children were still being followed up in pediatric and physical therapy departments. Children were excluded if they had a physical disability, diaphragmatic eventration, or were unable to perform all tests or procedures. Additionally, children with cardiac anomalies were excluded.

#### Allocation, Randomization, and Blinding

**Figure 1:**
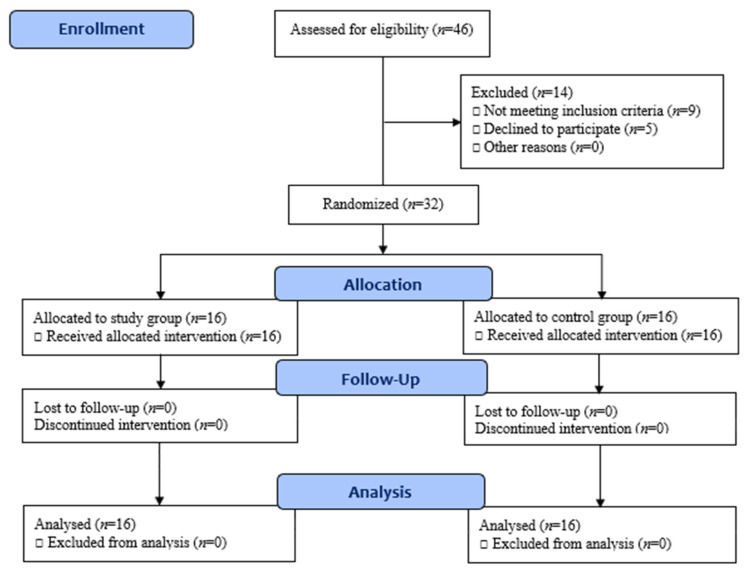
The CONSORT flow diagram of the study.

The children included in the study were randomly screened for eligibility according to the inclusion criteria of the study. Of the 46 children screened, 32 were enrolled, nine did not meet the inclusion criteria, and parents of five did not accept the invitation to enroll their children in the study. Random assignment was conducted before starting the study procedures with a 1:1 allocation ratio using consequent numerated occult envelopes. Allocation was performed by a blinded physiotherapist who was not included in the study objectives and procedures. The children were randomly allocated to study group (*n* = 16) and control group (*n* = 16). The CONSORT flow diagram is illustrated in Figure 1.

### 2.3. Sample Size Estimation

Using G*Power for Windows (V. 3.1.9.2, Dusseldorf, Germany), the sample size was determined in accordance with maximum inspiratory pressure (PImax) as a primary outcome measure. An unpaired *t*-test was performed to determine the power of 90% with α= 0.05, β = 0.95, and d = |0.81| obtained from a previous preliminary study of eight children, four per each group. Our study needed to enroll 26 participants in each of the two groups. The study, therefore, enrolled 32 children in each group to offset the anticipated 20% withdrawal rate.

### 2.4. Outcome Measures

A blinded physiotherapist who was not aware of the treatment assignment assessed respiratory muscle strength, lung function, and chest mobility (both pre- and post-treatment).

#### 2.4.1. Respiratory Muscle Strength

Respiratory muscle strength was evaluated using a POWERbreatheKH2 device that evaluates the strength index by measuring PImax, which was recorded in cmH_2_O. The children were asked to sit with their knees flexed at 90° and their noses closed with nose clips. Each child was asked to exhale to residual volume (RV) and then perform a maximal inspiratory effort sustained for 1–2 s. Three measurements were obtained, and the highest value was recorded [19]. The validity and accuracy of the POWERbreathe KH2 device for COPD patients have been demonstrated [20,21].

#### 2.4.2. Lung Functions

Lung functions were assessed by the Minispir^®^ (Rome, Italy) Light spirometer with Winspiro^®^ (Rome, Italy) Light software. Each child was seated with his/her knees flexed 90° and was asked to hold three deep breaths, take deep inspiration to total lung capacity (TLC), then exhale all the air inside the lungs to their residual volume (RV) to obtain the variables FEV1 (forced expiratory volume in 1 s) and FVC (forced vital capacity). Three measurements were obtained, and the highest values were recorded for analysis [22].

#### 2.4.3. Thoracic Excursion

Thoracic excursion was assessed by tape measurement, which is a reliable method for healthy individuals [23,24], patients with chronic obstructive pulmonary disease [25], and asthmatic children [26]. This measurement was performed at two levels: the axillary level (at the 4th intercostal space) and the thoracic level (at the tip of the xiphoid process). Each child was asked to stand in the upright position with hands over his/her head. Then, the 0 end of the tape was fixed on the midline of the body, while the other end was allowed to move. The tape should not be tight. The child was asked to breathe in and out maximally for both levels and hold the maximum inspiration or expiration for at least 2 s [27]. Axillary circumferences were recorded at maximum expiration and maximum inspiration. The discrepancy in circumferences between maximum expiration and inspiration was recorded (in cm) at the level of the 4th intercostal space. The same measurement was performed at the level of the xiphoid process. The thoracic excursion was presented as the mean of circumference discrepancies from the xiphoid and axillary levels. To avoid any errors and achieve interrater reliability, assessments were performed by two professional physiotherapists.

#### 2.4.4. Intervention

Children in both groups underwent 12-week usual chest physiotherapy in the form of bilateral vibration and gentle percussion for 3–5 min with distal finger phalanges to the upper apical lobes in modified drainage positions, placing the patient in a side-lying position or a prone position to increase oxygenation, at least 2–3 times a week [28,29].

However, the study group underwent a chest resistance exercise combined with chest expansion exercise in addition to the usual chest physiotherapy. For the chest resistance exercise, the children in the study group underwent sequential 12-week chest resistance and chest expansion exercises in three sessions per week. Manual resistance exercises and resistance exercises with the POWERbreatheKH2 were used to work out the chest in the past.

A manual chest resistance exercise was performed with the children in the side and supine lying positions by a professional physiotherapist by applying regular rhythmic resistance pressure on the coastal area during inspiration. The physiotherapist provided resistance to the inferior diaphragmatic contraction. Consequently, each child was instructed to take deep inspiration while gentle superior diaphragmatic pressure was being applied.

#### 2.4.5. The Power Breathe KH2 Resistance Exercise

Before starting the IMT protocol, simple instructions were provided to all children about the Power Breathe technique. After this first demonstration, however, the participants performed IMT by themselves. Each child was instructed to sit on a chair with their backs supported and shoulders relaxed, and then to put a nose clip securely over the nose, holding the device in his/her hand and putting the mouthpiece fully in his/her mouth so that the outer shield was between the lips and gums (the teeth on the inner shield). The training consisted of one set of 30 breaths. The first two breaths were unloaded; from the third to fifth breath, a load was gradually introduced to the child. Then, this pattern was repeated for six cycles. Each cycle consisted of 4 min of resistance exercise and 1 min of rest. The total duration of the exercise was about 30 min [30].

#### 2.4.6. Chest Expansion Exercise

With the child in a sitting or standing position, he/she was instructed to breathe in deeply while elevating both arms up and hold his/her breath for 2–3 s, then exhale and put his/her arms down. Then, while adducting the shoulders completely, the child took a deep breath in, held it for 2–3 s, and then exhaled. Finally, the child was asked to breathe in deeply while elevating one shoulder, hold for 2–3 s, and exhale while lowering one shoulder, then the second (repeating five times per exercise session).

### 2.5. Statistical Analysis

Data were analyzed using IBM SPSS Statistics for Windows (V. 26, IBM Corp., Armonk, NY, USA). The Shapiro–Wilk test was performed to examine the normal distribution of the data collected. The normally distributed variables were analyzed using an unpaired student’s *t*-test. However, the non-normally distributed variables were analyzed using Chi-square and Mann–Whitney tests for the baseline features. The differences between groups pre-and post-treatment with group × time interactions were determined using 2×2 repeated ANOVA. The Wilks’ lambda test was performed to calculate the *F*-value. A *p*-value ˂0.05 was considered the level of significance.

## 3. Results

As detailed in Table 1, no statistical difference was observed between groups in terms of baseline demographic and clinical characteristics (age, *p* = 0699; gender, *p* = 0.719; body mass index, *p* = 0.742; classification of CDH defect, *p* = 0.465; hospitalization period, *p* = 0.359; affected side, *p* = 0.694; and medical treatment, *p* = 0.414).

Using the 2×2 repeated ANOVA, significant time×group interactions in terms of lung functions were detected with better-predicted values for the study group, FVC (F= 4.82, CI 95% =−8.3, *p* = 0.005, and η^2^ = 0.16), FEV1 (F = 4.54, CI 95% = −7.4, *p* ˂ 0.001, and η^2^ = 0.14). Intragroup analysis post-treatment showed a significant improvement in the study group (FVC, *p* = 0.001; FEV1, *p* ˂ 0.001), whilst no statistical changes were observed in the control group (FVC, *p* = 0.265; FEV1, *p* = 0.172).

Regarding the PImax, significant time×group interactions were detected intergroup, supporting better values in the study group (F = 5.12, CI 95%= −10.5, *p* ˂ 0.001, and η^2^= 0.15). In intragroup analysis, post-treatment showed a significant improvement in the study group (*p ˂* 0.001), with no statistical changes in the control group (*p* = 0.411). For the thoracic excursions, significant time×group interactions were detected, with intergroup supporting better improvement in the study group (F = 4.31, CI 95% = −1.1, *p* = 0.036, and η^2^ = 0.17). In intragroup analysis, post-treatment showed a significant improvement in the study group (*p* = 0.022), with no statistical changes in the control group (*p* = 0.581), as detailed in Table 2.

## 4. Discussion

The current study was designed to investigate the effects of chest resistance exercise and chest expansion exercise on respiratory muscle strength, lung function, and chest mobility in children. The results show that when people perform chest resistance and chest expansion exercises, their lung functions (FVC and FEV1), respiratory muscle strength (PImax), and thoracic excursions improve.

In agreement with a previous study, our current study found that the number of males affected was higher than that of females (19 boys to 13 girls). Additionally, the majority of children had left-sided diaphragmatic hernia (23 left to 9 right) [5].

Congenital diaphragmatic hernia (CDH) is a life-threatening condition with long-term complications in survivors, such as recurrent respiratory tract infections, respiratory muscle weakness, and abnormal lung function during infancy and early childhood [31], so training programs were needed to study how their inspiratory muscle strength and lung function can be enhanced. In this respect, we investigated the effects of a 12-week chest resistance exercise program on inspiratory muscle strength, lung function, and thoracic excursion in children with post-operative congenital diaphragmatic hernia. The main findings of our study were that children with post-operative CDH who performed chest resistance exercise, chest expansion exercise, and respiratory muscle training did better than those who engaged in chest physiotherapy alone.

In both groups, there were significant improvements in inspiratory muscle strength, but the study group outperformed the control group. Increased respiratory muscle strength may be due to the exposure of inspiratory muscles to controlled load, which is regularly repeated, so there will be an increase in the sarcomere, an increase in muscle mass, and an increase in muscle ability to generate tension and strength. As the response of inspiratory muscles to resistance exercises is the same as that of skeletal muscles, they are considered the same as skeletal muscles functionally and morphologically [32]. Moreover, the increase in respiratory muscle strength after chest resistance exercises may be attributed to physiological adaptations induced by IMT: an increase in oxygen delivery, which may improve the aerobic capacity of the respiratory muscles and delay fatigue onset; hypertrophy of the diaphragm; an increase in type I fibers; and improvement in breathlessness [33].

To our knowledge, no researchers have previously studied the effects of chest resistance exercise and chest expansion exercises on inspiratory muscle strength, lung volume, and thoracic excursion in children with post-operative congenital diaphragmatic hernia. However, some researchers have used similar cases to evaluate the effect of chest resistance exercises. Lima et al. examined the impact of inspiratory muscle training on breathing distress in asthmatic children, and they concluded that inspiratory muscle training in conjunction with breathing exercises was effective in improving maximum inspiratory and expiratory pressure and improving exercise capacity with a consistent decrease in airway obstruction [34]. Additionally, Moawd et al. stated that inspiratory muscle training had a greater impact on improving inspiratory muscle strength in patients with obstructive sleep apnea [35]. Langer et al. studied the effects of inspiratory muscle training on patients with COPD, and they concluded that there were significant improvements in inspiratory muscle power output and improvement in breathing pattern [36].

Regarding lung function and thoracic excursion, there were significant improvements in both, and this may be attributed to a reduction in the muscle tension of the rib cage and an increase in its mechanical properties due to the movement of the rib cage. Decreased muscle tension is an important factor in increasing airflow during inspiration and expiration. Moreover, the thorax has an elastic structure that contracts and relaxes during breathing, and the expansion or contraction of the lungs is affected by the capacity of the thorax, which is increased by the increased elasticity of the skeletal muscle and surrounding soft tissue [37,38].

Our findings are supported by Hernández-Álvarez et al., who investigated the effect of 8 weeks of respiratory muscle training using threshold IMT on lung function and respiratory muscle strength in sedentary young people and discovered a significant change in FEV1, which was associated with a noticeable improvement in respiratory muscle strength [39]. Park also concluded that inspiratory muscle training in combination with rib cage mobilization is effective in improving chest wall movement, pulmonary function, chest expansion, and inspiratory muscle strength [40]. In addition, Rehman et al., found that passive stretching of the respiratory muscles can effectively benefit the status of such individuals, particularly in terms of chest expansion and functional capacity. Clinical and physical therapists might consider integrating passive stretching of respiratory muscles in the rehabilitation program because of the positive effects of muscle stretching and the fact that such an exercise is completely safe [41].

The main limitation of this study was the assessment with the POWERbreathe device, because this technique is dependent upon the effort and motivation of the child. Although careful instructions were given, the children were motivated, and assessments were performed three times. However, it was sometimes difficult to attain complete cooperation from all of the children. Expiratory muscle pressure was not analyzed in this study, so more studies in the future are needed to assess expiratory muscle strength. Additionally, our study could not confirm the long-term effect of the resistance exercises combined with the chest expansion exercises on children with a post-operative congenital diaphragmatic hernia. Therefore, future studies need to consider the follow-up effect 6–12 months after the end of treatment. Furthermore, future studies need to consider other age groups.

## 5. Conclusions

Chest resistance exercises combined with chest expansion exercises may improve respiratory muscle strength, lung function, and thoracic excursion in children with post-operative CDH. These findings suggest that children who have had CDH should perform chest resistance exercises and chest expansion exercises as part of their pulmonary rehabilitation programs.

## Figures and Tables

**Table 1:** Demographic and clinical characteristics.

Characteristics	Control Group (*n* = 16)	Study Group (*n* = 16)	*p*-Value
Age, years	12.2 ± 1.4	12.4 ± 1.5	0.699
Gender, m/f	9/7	10/6	0.719
Body mass index, Kg/m^2^	20.8 ± 3.5	21.2 ± 3.3	0.742
Hospitalization period, months	2.1 ± 0.5	1.9 ± 0.7	0.359
Classification of CDH defect, *n* (%)			
B-defect	9 (56.25)	11 (68.75)	0.465
C-defect	7 (43.75)	5 (31.25)	
Affected side, *n* (%)			
Left side	11 (68.75)	12 (75)	0.694
Right side	5 (31.25)	4 (25)	
Medical TTT, *n* (%)			
Inhaled corticosteroid	3 (18.75)	5 (31.25)	0.414
Inhaled corticosteroid+ long-acting β2-agonist	13 (81.25)	11 (68.75)	

Significant difference at *p* ˂ 0.05; CDH: congenital diaphragmatic hernia; TTT: treatment.

**Table 2:** Pre- and post-treatment differences intragroup and intergroup.

Measures	Control Group (*n* = 16)	Study Group (*n* = 16)	Mean Difference (95% CI)	Group × Time Interaction	
				*p*-Value	η^2^
FVC, pred.					
Pre-	77.2 ± 10.7	77.7 ± 11.2	−0.5 (−8.41 to 7.41)	0.005	0.16
Post-	81.3 ± 10.5	89.6 ± 9.8	−8.3 (−15.6 to −0.97)		
*p*-value	0.265	0.001			
FEV1, pred.					
Pre-	70.6 ± 7.4	70.8 ± 7.8	−0.2 (5.7 to 5.3)	˂0.001	0.14
Post-	74.1 ± 7.2	81.5 ± 5.4	−7.4 (−11.99 to −2.8)		
*p*-value	0.172	˂0.001			
PImax, cmH_2_O					
Pre-	39.4 ± 7.8	39.8 ± 8.1	−0.4 (−6.14 to 5.34)	˂0.001	0.15
Post-	41.2 ± 7.6	51.7 ± 6.8	−10.5 (−15.71 to −5.3)		
*p*-value	0.411	˂0.001			
Thoracic excursions, cm					
Pre-	6.5 ± 1.3	6.7 ± 1.5	−0.2 (−1.21 to 8.1)	0.036	0.17
Post-	6.7 ± 1.4	7.8 ± 1.2	−1.1 (−2.04 to −0.16)		
*p*-value	0.581	0.022			

All data are displayed as mean ± SD; FVC: forced vital capacity; FEV1: forced expiratory volume in 1 s; PImax: maximal inspiratory pressure; η^2^: Eta square.

## Data Availability

The authors declare that all relevant data supporting the findings of the study are available within the manuscript.
